# Successful Anesthetic Management of an Adult with Sotos Syndrome

**DOI:** 10.1155/2022/2585015

**Published:** 2022-02-24

**Authors:** Andrew Winegarner, Mark C. Kendall, Harish Lecamwasam

**Affiliations:** Department of Anesthesiology, Rhode Island Hospital, The Warren Alpert Medical School of Brown University, Providence, Rhode Island 02903, USA

## Abstract

Sotos syndrome is a rare genetic disorder presenting with craniofacial abnormalities, profound hypotonia, and cardiac abnormalities, giving rise to several potential challenges and concerns for an anesthesiologist. When preparing for a Sotos syndrome patient's case, we consulted the literature for precedents on how to plan the anesthetic, to which we were only able to find a few reports and nothing in the age group our patient fell within. We present our case of an adult in addition to examining the previous cases so as to document a precedent when encountering patients with this syndrome in the operating room. We describe a unique case of a nonverbal adult with hypotonia and severe craniofacial abnormalities who successfully underwent multiple dental extractions under general anesthesia, with no complications other than a delay of emergence attenuated by naloxone. Our case and the seven previous documented cases over the past several decades demonstrate anesthesia, including paralytics and intubation itself safe despite obvious concerns given the common features of the syndrome for both pediatric patients and the one adult we described in this report.

## 1. Introduction

Sotos syndrome, also known as cerebral gigantism, is a rare genetic disorder with an estimated prevalence at birth of 1 in 14,000 [1]. An overwhelming majority of cases are linked to mutations in the NSD1 gene located on chromosome 5 [2]. Children with this syndrome typically have distinctive facial features, macrocephaly, learning disabilities, and advanced bone age. Characteristic facial features include frontal bossing, prominently long narrow chin, and downslanting palpebral fissures [3]. Patients also present with hypotonia, cardiac abnormalities, scoliosis, renal anomalies, and behavioral problems compounding the learning difficulties. As children with Sotos syndrome age, their physical abnormalities can become more subtle. Adults with Sotos syndrome can manifest a wide spectrum of disease where some are completely dependent on care givers while others are able to live independently [3].

We present a case of an 18-year-old male with Sotos syndrome who had multiple dental extractions under general anesthesia and summarize previously published cases of Sotos syndrome anesthetics. In searching the literature, we found seven case reports describing anesthesia in children with Sotos syndrome [4–10]. However, we were not able to find any reports on the anesthetic management of adults with Sotos syndrome. As Sotos syndrome patients age, it is worth revisiting their anesthetic management and documenting any precedents available. Authors obtained permission and written informed consent from the patient's mother presented in this case report.

## 2. Case Presentation

An 18-year-old male presented for dental restorations and extractions. His medical history included Soto syndrome, Guillain–Barre syndrome, epilepsy, scoliosis, stage III chronic kidney disease (associated with a solitary dysplastic kidney), and autism spectrum disorder. Home medications included sertraline, risperidone, gabapentin, divalproex, diazepam, benztropine, fluticasone, and an albuterol inhaler, and no history of any surgical procedures. A 2-year-old transthoracic echocardiogram was unremarkable. Physical exam revealed an elongated mandible, frontal bossing, significant hypotonia and kyphosis. He was nonverbal, agitated, and uncooperative, attempting to hit preoperative nurses. Due to COVID-19, his mother was unable to accompany and console him en route to the operating room. Because the patient was nonverbal, the mother signed consent on the patient's behalf for publication of the case.

He was premedicated with 15 mg of midazolam via a gastric tube prior to transport to the operating room, which sufficiently placated the agitation. In the operation room, standard monitors were placed and anesthesia induced using nitrous oxide and sevoflurane via the mask without difficulty. Following induction, peripheral intravenous access was established, and propofol 50 mg and rocuronium 30 mg were administered in preparation for intubation. Direct laryngoscopy was performed using a Miller 2 blade. A Cormack-Lehane grade 1 view was obtained on the first attempt, and a 6.0 endotracheal tube was placed without difficulty. Anesthesia was maintained with an oxygen/air mixture and sevoflurane, and a single dose hydromorphone 0.5 mg was administered intravenously at procedure start.

The patient's intraoperative course was unremarkable. No additional neuromuscular blocking agent or narcotic was administered. At the procedure end, sugammadex 200 mg IV was administered after a train-of-four assessment demonstrated 1/4 twitches with significant fade. Despite full reversal of neuromuscular blockade (4/4 twitches without fade) and an end-tidal sevoflurane concentration of 0.3%, the patient remained unresponsive and without spontaneous respiratory effort. Following three doses of Narcan 0.4 mg intravenously, the patient had return of spontaneous respirations with good tidal volumes and rate. Patient was extubated without incident and transported to the postanesthesia care unit on a non-rebreather mask. The patient had an unremarkable postoperative course and was discharged to home 60 minutes later.

## 3. Discussion

Sotos syndrome has a number of phenotypes that could be of concern to an anesthesiologist. These include craniofacial abnormalities, muscular hypotonia, behavioral disorders, and potential cardiac and renal abnormalities. Before discussing an anesthetic plan, a literature review was done to look for precedents given the concerning features of Sotos syndrome, as the syndrome was unknown to us before taking care of this patient. Our patient manifested more severe diseases with significant scoliosis, hypotonia, kyphosis, and a single dysplastic kidney. He was also nonverbal and had a history of aggressive behavior. An airway exam was not possible due to agitation, so adjuncts to manage a difficult airway were readily available such as supraglottic airways, video-laryngoscopy, fiberoptic bronchoscope, and bougies in the event that the craniofacial abnormalities provided a difficult airway. However, the patient had an easy mask airway and easy intubation with direct laryngoscopy. This is consistent with the experience of other authors in the management of airways in children with Sotos syndrome [4–10], wherein there was no difficulty with intubation. Despite preexisting hypotonia, we also did not observe any excessive sensitivity to nondepolarizing neuromuscular blockade or delay in return of neuromuscular function following administration of sugammadex. This is also consistent with published reports in the pediatric population [4, 7, 8], wherein there were no issues with neuromuscular blockade despite pronounced hypotonia.

Our review of the literature revealed case reports describing anesthesia in children with Sotos syndrome [5–10], none of which had any anesthetic complications, all summarized in Table 1. This is being the 8th such report without any major incident with regard to hypotonia or airway. Regardless, we recommend close train-of-four monitoring and use of reversal agents as appropriate. We also recommend careful titration of opioids given the potential for sensitivity. Given the rarity of the syndrome, we hope this report will provide a precedent for the anesthetic management of an adult with Sotos syndrome and a means to quickly see previously documented anesthetic management.

## Figures and Tables

**Table 1:** Summary of previously documented Sotos syndrome anesthetic management.

Year	Author	Age	Weight (kg)	Sex	Surgery	Premedication	Induction	Anesthetic	Paralytic	Analgesia	Airway	Intraoperative complications
2021	Winegarner et al.	18	41	M	Dental extractions and restoration	Midazolam 15 mg (gastric tube)	Mask, 50% N_2_O, 8% sevo, propofol 1.2 mg/kg	Sevoflurane	Rocuronium 0.7 mg/kg	Hydromorphone 12 mcg/kg	ETT 1st attempt, grade 1 view	Delayed emergence attenuated with naloxone
2017	Chung et al.	4	19.4	M	Hydrocelectomy	Glycopyrrolate 100 mcg IM	Thiopental 5 mg/kg	Sevoflurane	Rocuronium 0.3 mg/kg	Fentanyl 1 mcg/kg	ETT 1st attempt, grade 1 view	None
2011	Chierichini et al.	7	NA	M	Flat foot surgery	None	Sevoflurane	Regional	None	Sciatic nerve block with 10 ml of ropivacaine 0.5%	NA	None
2003	Adhami et al.	2.5	17	M	Inguinal hernia repair	Atropine 11.8 mcg/kg	Mask, 50% N_2_O, 8% sevo	N_2_O and sevoflurane	None	NA	ETT 1st attempt, grade 1 view	None
2003	Adhami et al.	1.5	14.8	M	Inguinal hernia repair	NA	NA	NA	Cisatracurium 0.2 mg/kg	Morphine 68 mcg/kg	ETT 1st attempt, grade 1 view	None
2001	Varvinski et al.	13	55	F	Tibia repair	NA	Thiopental 5.45 mg/kg	Sevoflurane	Succinylcholine 1.81 mg/kg	Fentanyl 1.36 mcg/kg	ETT 1^st^ attempt, grade 1 view	None
1993	de Nadal et al.	13	80	M	Parietal bone resection	Atropine 10 mcg/kg	Thiopental 5 mg/kg	N_2_O and Isoflurane	Atracurium 0.5 mg/kg	Fentanyl 2 mcg/kg	Nasal intubation failed, 2nd attempt with oral ETT	None
1993	de Nadal et al.	1	10.6	M	Brain tumor resection	Atropine 10 mcg/kg	Thiopental 5 mg/kg	N_2_O and isoflurane	NA	Fentanyl 1 mcg/kg	Nasal intubation	None
1991	Suresh	14	90	M	Spinal fusion	Diazepam 10 mg, oral	Halothane	N_2_O and halothane	Vecuronium 0.1 mg/kg	Meperidine	ETT	None
1991	Suresh	14	NA	M	Spinal fusion revision	NA	Thiopental	N_2_O and enflurane	Succinylcholine and vecuronium	Papaveretum	NA	None
1991	Jones et al.	NA	15.6–25.8	NA	Hernia repairs, Harrington rods	Trimeprazine	Halothane, enflurane, or thiopentane	Isoflurane	Atracurium and alcuronium	Morphine and fentanyl	ETT without incident on each occasion	None

ATT, attempt; ETT, endotracheal tube; NA, not specified in the article; NI, nasal intubation; UGSNB, ultrasound-guided sciatic nerve block. Doses are specified in the table if they were provided in their respective reports. The Adhami et al. 2003 report described two anesthetics given to the same patient at 18 months and 30 months old. The Suresh et al. 1991 report describes two anesthetics given to the same patient separated by a week. The Jones et al. 1991 report described five anesthetics given to one patient over a period of three years.

## Data Availability

The data used to support the findings of this case report are included within the article.
